# ETHNIC DIFFERENCES IN FACTORS INFLUENCING ATTITUDES TOWARD SUICIDE AND PHYSICIAN-ASSISTED SUICIDE AMONG ELDERS

**DOI:** 10.1093/geroni/igz038.2204

**Published:** 2019-11-08

**Authors:** Ruben Martinez

**Affiliations:** Michigan State University, East Lansing, Michigan, United States

## Abstract

This study examines differences in attitudes toward suicide and physician-assisted suicide in chronic pain scenarios among Latino and White elders. Face-to-face interviews were conducted with 204 elders at four community-based, outpatient care sites in the metropolitan area of San Antonio, Texas. In addition to ethnicity and the suicide-related questions, the interviews collected data on age, gender, marital status, education, income level, acculturation (Latinos only), depression, self-reported health status, daily living functioning, and religiosity/spirituality. In general, there were no ethnic group differences in attitudes, however, the factors associated with those attitudes varied between ethnic groups. Among Whites, attitudes toward suicide and toward physician-assisted suicide were significantly and negatively associated with religiosity. Among Latinos, depression was significantly and positively associated with attitudes toward suicide in chronic pain scenarios, while acculturation was significantly and negatively associated with attitudes toward physician-assisted suicide in chronic pain scenarios. This study’s findings suggest that depression among Latino elders and religion among White elders are determinant factors of attitudes toward suicide in chronic pain scenarios. Future research is needed to confirm our findings with a more heterogeneous study sample, including Latinos from different countries of origin (e.g. Mexican Americans, Puerto Ricans, Cubans); and more heterogeneous ethnic groups in terms of socioeconomic status and educational level characteristics.

